# Constipation and clozapine: a QI project in Leicestershire Partnership NHS Trust, (LPT)

**DOI:** 10.1192/bjo.2021.248

**Published:** 2021-06-18

**Authors:** Ehimwenma Evbuomwam, Dan Kinnair, Mohammad Mirza, Julian Coleman

**Affiliations:** 1 Leicester Partnership Trust; 2 Leicestershire Partnership Trust, University of Leicester

## Abstract

**Aims:**

Constipation in patients on Clozapine is the biggest cause of mortality. We have no set protocol in LPT for how to manage and monitor Constipation in Clozapine initiation in the inpatient setting. Internationally protocols, (such as the Porirua protocol) exist but have not been widely used locally.

We wanted to assess local compliance with monitoring constipation in patients admitted to hospital and started on Clozapine. We also wanted to assess whether patients are prescribed PRN or regular laxatives, before considering implementing a local protocol.

**Method:**

In LPT we use the ZTAS system for prescribing Clozapine. They provided us with a list of patient IDs who had recently started on Clozapine.

We captured data on patients started on Clozapine.

1. What date was this started?
2. What date was either PRN or regular laxatives started?
3. Was a bowel chart recorded?
4. Any evidence of constipation or significant bowel issues relating to Clozapine?

**Result:**

We initially analysed 30 patients, (20 of whom were initiated on Clozapine as inpatients, and 10 as outpatients). A bowel chart was started in only 1 inpatient. Laxatives were started in 50% (15, only 3 of whom were outpatients). 14 were regular and 1 was a PRN prescription. 12 inpatients had constipation, and 1 outpatient suffered with constipation. 2 patients suffered with diarrhoea but there were no other significant issues with bowel problems.

**Conclusion:**

From our initial data we can see that there are many inconsistencies in practice.

Existing patients on Clozapine attend a local clinic, (Clozapine clinic) where ongoing monitoring of constipation, (and other parameters, e.g. ECGs etc are completed).

We have written a new protocol which we will share, that the trust has implemented, that identifies when PRN and regular laxatives should be prescribed. We have also expanded the protocol to agree for initiation of Olanzapine bowel charts and PRN laxatives should be used.

